# Measure and characterization of the forces exerted by growing multicellular spheroids using microdevice arrays

**DOI:** 10.1371/journal.pone.0217227

**Published:** 2019-05-23

**Authors:** Laurene Aoun, Stanislas Larnier, Pierre Weiss, Martine Cazales, Ariane Herbulot, Bernard Ducommun, Christophe Vieu, Valérie Lobjois

**Affiliations:** 1 ITAV, Université de Toulouse, CNRS, UT3, Toulouse, France; 2 CNRS, LAAS, Toulouse, France; 3 Université de Toulouse, LAAS, Toulouse, France; 4 CHU de Toulouse, Toulouse, France; 5 Université de Toulouse, INSA, Toulouse, France; Rensselaer Polytechnic Institute, UNITED STATES

## Abstract

Growing multicellular spheroids recapitulate many features of expanding microtumours, and therefore they are an attractive system for biomechanical studies. Here, we report an original approach to measure and characterize the forces exerted by proliferating multicellular spheroids. As force sensors, we used high aspect ratio PDMS pillars arranged as a ring that supports a growing breast tumour cell spheroid. After optical imaging and determination of the force application zones, we combined 3D reconstruction of the shape of each deformed PDMS pillar with the finite element method to extract the forces responsible for the experimental observation. We found that the force exerted by growing spheroids ranges between 100nN and 300nN. Moreover, the exerted force was dependent on the pillar stiffness and increased over time with spheroid growth.

## Introduction

Sensing compression and tension forces (i.e., mechanosensing) is an important component of cell physiology. Changes in mechanical homeostasis within tissues are often observed during tumour growth. Solid tumour growth is associated with stiffening of the tumour tissue due to cell proliferation and modification of the extracellular matrix components. Such tissue stiffening involves the generation of mechanical forces that accumulate within the growing tumour and that, in turn, are applied on and deform the surrounding tissue [1, 2]. Besides genetic alterations and biochemical signals, these mechanical forces also contribute to tumour progression and resistance to treatment. Several reports show that changes in mechanical properties can modulate tumour cell behaviour by influencing their proliferation, migration and invasion properties [3–9]. Moreover, Jain and collaborators demonstrated that these mechanical forces also induce vessel compression and increase the interstitial fluid pressure, ultimately affecting drug delivery [10]. Despite their crucial role, only few experimental methods are available to measure the forces generated by growing solid tumours or by multicellular tumour spheroids, an in vitro model that recapitulates the 3D tumour organization in vitro [3, 10, 11]. Moreover, it is still not clear how the forces generated by growing solid tumours vary depending on the microenvironment stiffness and over time.

Inspired by the strategy of arrays of discrete microfabricated pillars (posts) of silicone elastomer to measure forces exerted by single cells [12, 13], we previously developed a technological process for fabricating high aspect ratio polydimethylsiloxane (PDMS) micropillars (300μm in height) with different diameters adapted to the characterization of the mechanical interactions between a growing multicellular tumour spheroid and its environment. The microdevices are made of 300 μm high cylindrical PDMS micropillars, arranged in a circular manner around the spheroid [14]. Using time-lapse video-microscopy, we demonstrated that pillar displacement induced by growing spheroids depends on pillar stiffness. This suggests that during spheroid growth, cells collectively generate forces that could be measured using these pillars as force sensors. This paper is devoted to the implementation of this technique and describes in details how the force modulus can be extracted from 3D pillar displacement measurements. It also investigates the force temporal changes during spheroid growth as a function of the micropillar stiffness.

Traction force determination in single-cell experiments with microfabricated pillars relies on measuring the pillar top displacement [12, 13]. However, multicellular spheroid growth induces large pillar deformations, and measuring the force based only on pillar top deflection would not be accurate. In some studies, pillar deflection was evaluated by volumetric imaging [15, 16]. Lemon and collaborators demonstrated, by using a finite element model (FEM) analysis of pillar deflection, that top displacement does not allow precise force measurements for deflections larger than 4 μm. By imaging the entire pillar (from top to base), they showed that considering the whole pillar deflection pattern provides a more accurate analysis of single-cell traction forces than just the comparison of the top position before and after deflection[16]. With the aim of measuring the forces generated by multicellular tumour spheroids relative to the microenvironment stiffness and during spheroid growth, we developed a methodology to estimate forces through the volumetric analysis of the growing spheroid-induced 3D deflection of high aspect ratio micropillars with different spring constants. This methodology is based on 3D imaging and reconstruction of the micropillars and then on comparison of the experimental results with the data derived from a FEM analysis of 3D pillar deflection. We show that spheroid cells collectively apply higher forces on pillars of increasing stiffness and that these forces increase progressively with spheroid growth.

## Materials and methods

### Fabrication of the PDMS microdevices

The master mold was fabricated using UV photolithography on thick SU-8 3050 photoresist (MicroChem Inc.) of 300 μm, spin coated on a 4-inch silicon wafer. The photoresist was exposed to UV light via the mask that contains circular designs of the pillar shape. The unexposed parts of this negative photoresist were dissolved in the development solution PGMEA, which creates cylindrical holes that serve as a master mold for the PDMS replica. After development and hard bake, the SU-8 mold was treated anti-adhesively with octadecyltrichlorosilane (OTS) in liquid phase to prevent PDMS adhesion to the mold. The PDMS pre-polymer was mixed with the polymerization agent Sylgard 184 (10:1 ratio), poured on the SU-8 mold and cured at 80°C for 5 hours. The PDMS replica could then be easily cut and gently unmoulded. Microdevices fabrication was described in detail in our previous publication [14].

### Measurement of Young’s modulus of PDMS material

Measurement of the Young’s modulus relied on the compressive force technique, using the ElectroForce 3100 Bose instrument and the WinTest software. The measurement was done on dimensionally calibrated PDMS macroscopic posts (height = 10 mm, and diameter = 9 mm) made in the same polymerization conditions as the micropillars. The instrument has a maximum force capacity of ±22 N. The post was compressed by 10% of its height at a compression speed of 1mm/sec. This technique gives the compression force and the dimensional changes of the PDMS post, allowing the Young’s modulus measurement. In this compression experiment, the linear elasticity theory gives a straightforward relation between the applied force and the deformation, supporting the hypothesis of uniaxial stress:

$$\frac{F}{S}\;=\;E\times \frac{\Delta L}{H}$$

where F is the compression force, ΔL is the compressed deformation, S is the post section (= Π r^2^), r is the post radius, and H is the post height.

From the compression data, the slope can be calculated as $\frac{F}{\Delta L}$, and the post height and diameter can be measured by optical microscopy. The results obtained on our samples gave a value of E = 2.6 ± 0.2 MPa.

### Spheroid models

MCF7 mammary cancer cells (ATCC) were cultured in RPMI 1640 medium (Invitrogen, France) supplemented with 1μM insulin (Sigma), 10% foetal calf serum (FCS) (Invitrogen, France), and 1% penicillin/streptomycin in a humidified atmosphere of 5% CO2 at 37 °C. Spheroids were prepared in Dulbecco’s Modified Eagle’s Medium/Nutrient Mixture F-12 (DMEM F-12) (Invitrogen, France), supplemented with 5% FCS, 1% penicillin/streptomycin, 10^−8^ M 17β-oestradiol (Sigma), 1 μM insulin, 20 ng/ml epidermal growth factor (Invitrogen) and B-27 Supplement (1X, (Invitrogen) in a humidified atmosphere of 5% CO_2_ at 37 °C. A concentration of around 1000 cells in 100μl was loaded in each well of poly-HEMA coated 96-well plates. Plates were centrifuged at 600 g for 6min, and then incubated in a humidified atmosphere of 5% CO2 at 37 °C. After 3 days, spheroids of about 300μm in diameter were recovered from each well and transferred one by one to a PDMS microdevice by direct micropipetting or using microtweezer.

### Pillar fluorescent staining and confocal imaging

Unmolded PDMS chips were stained with 25 μg/ml 1,1′-dioctadecyl-3,3,3′,3′-tetramethylindocarbocyanine perchlorate (DiI), a fluorescent lipophilic tracer, in phosphate buffer solution (PBS). The PDMS microdevices were covered with the staining solution at room temperature for 20min, and then washed three times for 5min to remove excess staining. Then, culture medium was added and spheroids were deposited in the centre of the microdevices.

Confocal acquisitions were done using Zeiss LSM 510 NLO laser-scanning microscope, fitted with a water immersion 20X objective.

### 3D reconstruction algorithm

The algorithm works by sequentially segmenting each pillar, slice by slice. The general outline is as follows:

- Segmentation of the pillars bases in 2D using the first slice of the 3D stack image.
- From the second to the last slice: the information from the previous slice is used to define a search region and to segment the current slice.
- Smoothing the pillar skeletons to reduce segmentation defects.

The algorithm outputs are the pillar centre and radius of each slice as well as a confidence index given by the energy [17]. A detailed description of the algorithm is given in the Supporting information.

### COMSOL simulation

The commercial finite element software COMSOL 4.3 was used for the 3D simulation of micropillar mechanical deformation. This software solves equations of linear elasticity. A PDMS pillar was modelled as a 3D elastic cylinder with a Young’s modulus E = 2.6 MPa, a Poisson’s ratio of 0.499, and a density ρ of 927 Kg/m^3^. The pillar height was always 300μm and the diameter changed according to the diameter of pillars of the experimental data (28 and 36 μm). The modelled pillar was discretized into tetrahedral mesh elements with a predefined element size: Fine (minimum and maximum element size: 3 and 24 μm, maximum element growth rate: 1.45, resolution of curvature: 0.5, and resolution of narrow regions: 0.6). The applied boundary force was horizontal, unidirectional and distributed uniformly on the upper 175μm of the micropillar, as determined experimentally.

Three parameters were computed to identify the simulated curve that best fitted the experimental curve for each 3D reconstructed pillar: the sum of squares of residuals SS_res_, the coefficient of determination R^2^, and the largest shift M. The following equations were used:

for one pillar, let *d*_*i*_ and *v*_*i*_ indicate the detected and simulated displacement at slice *i* ∈ {1, …, *n*_*z*_}, respectively,

$$SS_{res}\;=\;\sum_{i\;=\;1}^{n_{z}}{\left(d_{i}-v_{i}\right)}^{2}$$

$$R^{2}\;=\;1-\frac{{SS}_{res}}{{SS}_{tot}}\;with\;SS_{tot}\;=\;\sum_{i\;=\;1}^{n_{z}}{\left(d_{i}-\overset{-}{d}\right)}^{2}\;and\;\overset{-}{d}\;=\;\frac{1}{n_{z}}\sum_{i\;=\;1}^{n_{z}}d_{i}$$

$$M\;=\;\underset{i\in \{1,\ldots ,n_{z}\}}{\text{max}}\;\left|d_{i}-v_{i}\right|$$

## Results

### Principle of the measurement of the forces induced by growing spheroids

**Fig 1:**
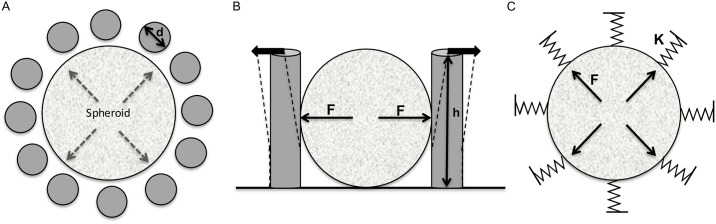
High aspect ratio microdevices for the measurements of forces exerted by growing spheroids. (A) Plane view of the circular arrangement of the PDMS pillars surrounding a spheroid. (B) Lateral view of the pillar displacement induced by a growing spheroid. (C) Forces (F) generated by the growing spheroid on the surrounding environment induce an outward displacement of the pillars. Pillars are defined by their diameter (d), height (h), and spring constant (K).

**Fig 2:**
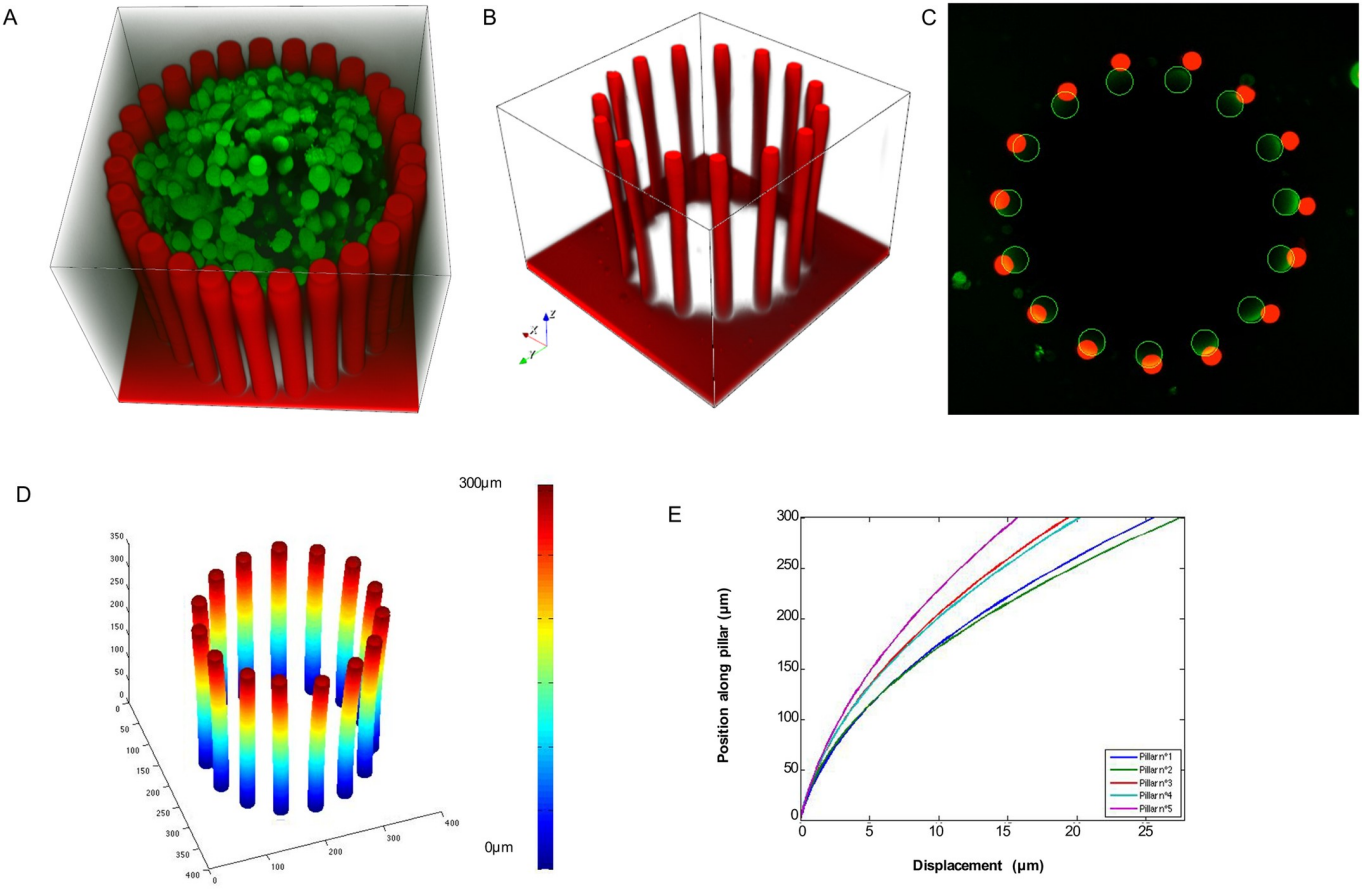
3D reconstruction of pillar deformations. (A) 3D confocal image showing a microdevice stained with Dil (red; fluorescent lipophilic tracer) surrounding an MCF-7 (mammary cancer cell line)-derived spheroid stained with the fluorescent dye CMFDA-SE (green). (B) 3D visualization of the z-stack images (step size = 4μm) of the microdevice where pillar deflection induced by the spheroid growth (unlabelled spheroid) is clearly visible. (C) Superimposition of the first (base) and last (top) images of the z-stack showing the pillar radial displacement; green, bottom, and red, top of each micropillar. (D) 3D reconstruction of the detected centres of all pillars in each image of the z-stack (75 images). (E) Deformation curves of five pillars showing the deflection magnitude along the pillar length (300 μm).

We previously developed biocompatible ring-shaped microdevices composed of arrays of high aspect-ratio flexible PDMS pillars that can be used as force microsensors to investigate the mechanical forces of multicellular tumour spheroids[14]. The principle of the mechanical model used to measure the forces exerted by a growing spheroid is schematically illustrated in Fig 1. By considering the pillars as force sensors, we could determine the force on the basis of their radial displacement. Therefore, each pillar was characterized by its geometrical and material parameters: diameter (d), height (h) and spring constant (K). We then used these parameters and the deflection amplitude to model the pillar displacement, and to calculate the forces (F) responsible for such deflection. This model allowed linking the pillar displacement to the collective forces induced by spheroid growth. To evaluate the force exerted by a 3D cohesive cell population, we used spheroids made of MCF-7 mammary tumour cells because they maintain a coherent 3D organization and grow homogeneously inside the microdevice, thereby inducing a nearly radial displacement of all the microdevice pillars (Fig 2 and S1 Movie).

### Volumetric imaging and 3D reconstruction of the deformed microdevices

Measuring the forces generated by growing spheroids requires first to experimentally determine the pillar deflection by volumetric analysis. To this aim, we acquired 4μm step-size z-stacks of growing spheroids within the microdevices by 3D imaging using confocal microscopy (Fig 2A). To visualize the entire pillar length, we stained them with the fluorescent lipophilic tracer DiI (Fig 2A and 2B). The images were acquired using a straight confocal microscope; the bottom of the pillars is 300μm away from the top, and slightly more from the objective, thus making the image intensity variable along the z axis. The apparent size difference between the top and the bottom of the pillars is a consequence of image degradation due to several optical defects, such as scattering and z-anisotropy. To determine the pillar deflection from the confocal images and to measure the 3D deformations, we developed a specific pillar reconstruction routine in MATLAB. This segmentation tool takes into account technical issues, such as optical degradation or altered pillar shape on images, and produces the skeleton of each pillar as well as its radius at different heights (Fig 2D). We could apply this segmentation technique also for complex pillar deformations caused by spheroids that did not induce a radial pillar displacement because their cells aggregated around some of the pillars (see the example of GM637-derived spheroids in Fig C in S1 File). The algorithm is described in detail in Supporting Information. From the 3D analysis, we calculated the deflection curves for each pillar based on the x,y coordinates of the pillar centre along the 300 μm of its length (Fig 2D and 2E). This analysis confirmed that MCF-7 cell spheroids induce a radial displacement of the pillars (Fig 2C).

### Measurement of the forces exerted by growing spheroids

**Fig 3:**
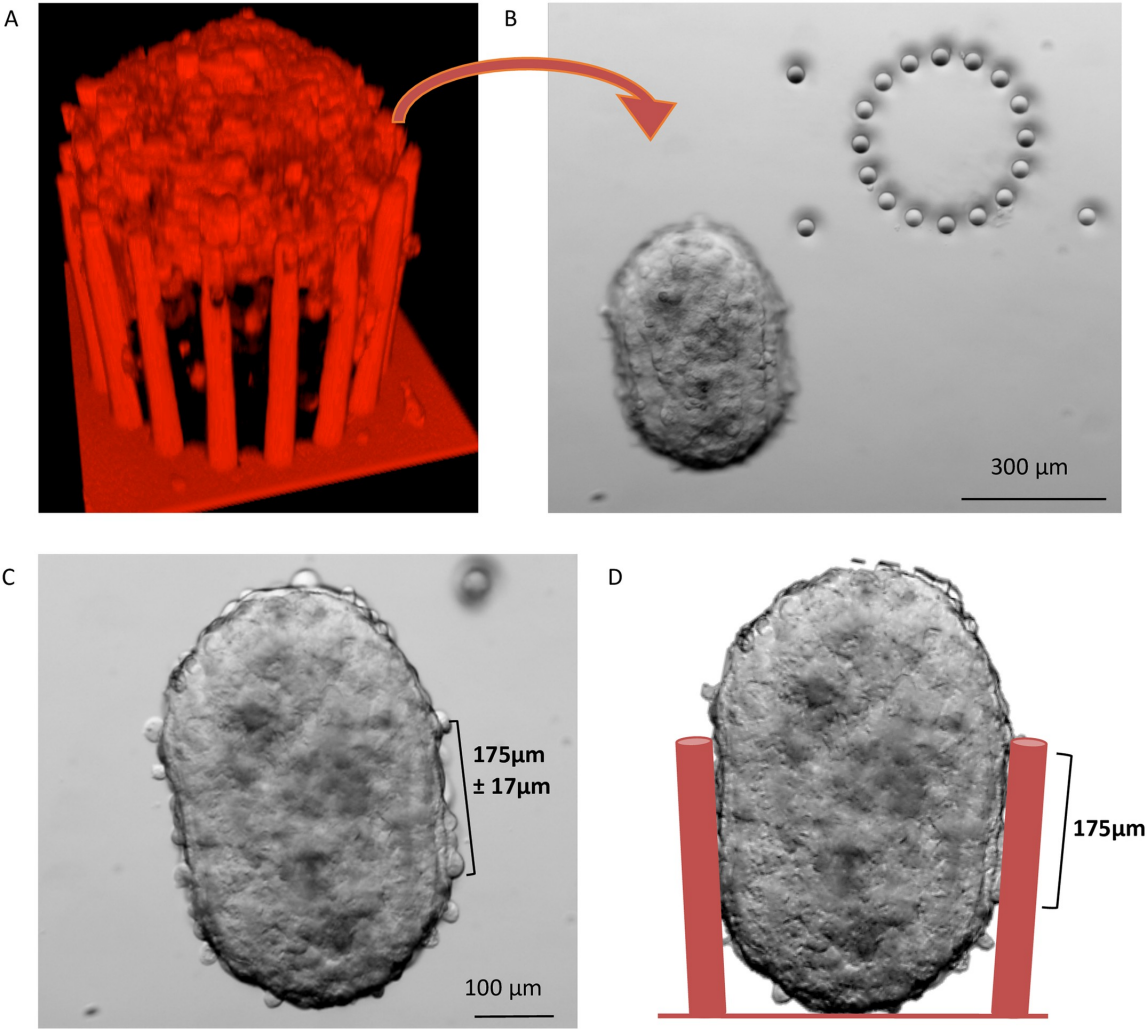
Determination of the spheroid-microdevice contact zone. (A) A 3D confocal image of a stained living spheroid in the microdevice showing the imaging limitations to access the whole spheroid. (B) Bright field microscopy image taken directly after the spheroid removal from the device showing the pillar imprints (arrowheads) on the spheroid. (C) Bright field image of the spheroid showing the round bottom and top and the very straight borders that define the contact zone, which was found to be 175±17 μm long. (D) Sketch of the spheroid in the microdevice showing the contact zone at day 4.

**Fig 4:**
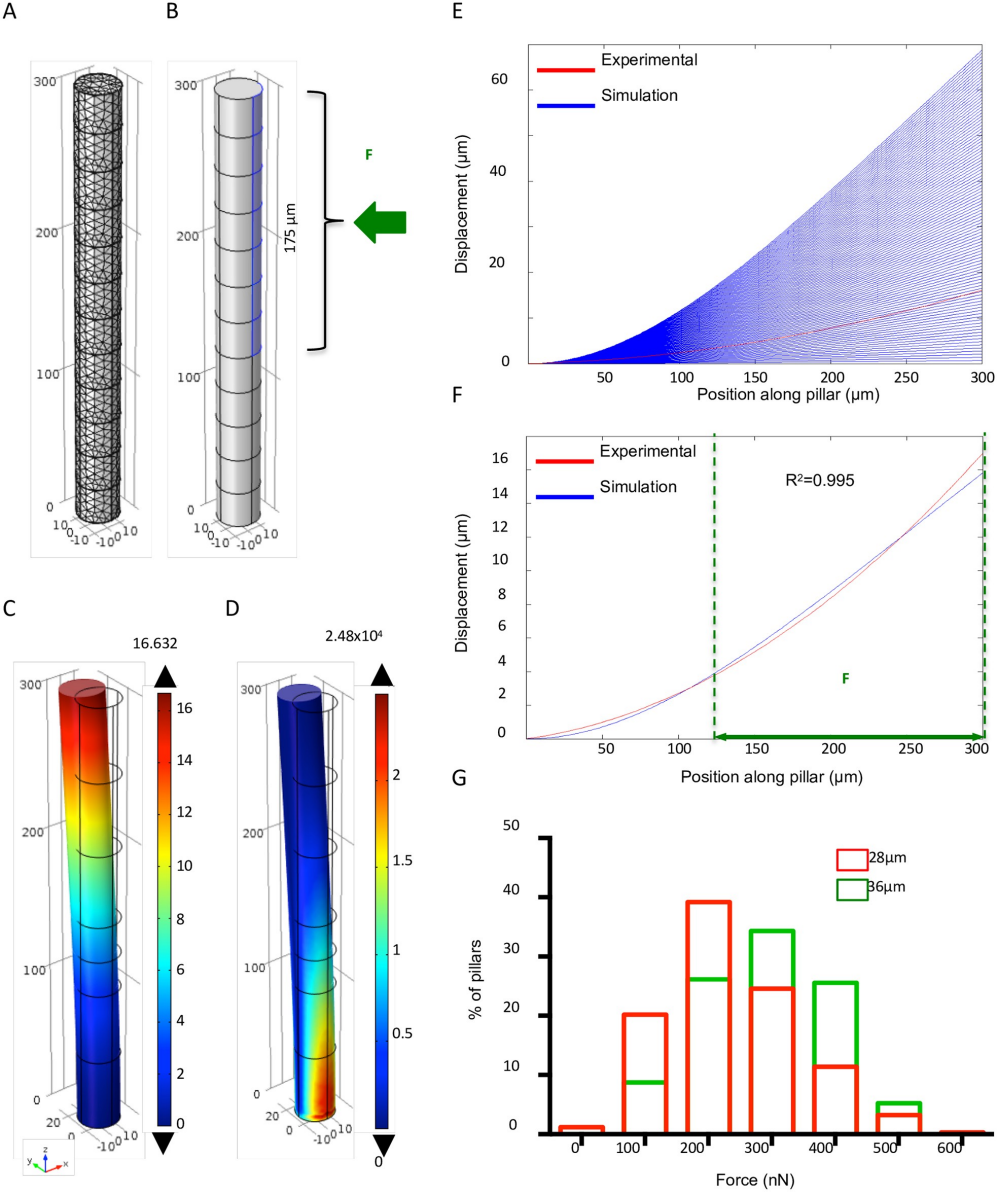
Determination of the forces exerted by growing spheroids based on pillar deflection using the finite-element method analysis. (A) Fine tetrahedral meshing of the whole micropillar. (B) 3D model of the cylindrical micropillar with the introduced geometrical and material parameters. The uniform unidirectional boundary of the force (F) load on the upper 175 μm of the pillar length is highlighted in blue. (C) Pillar deflection: the highest deformation is at the pillar top (colour code unit: μm). (D) Von Mises stress for the deformed pillar showing the highest stress at the fixed bottom (colour code unit: N/m^2^). (E) Abacus of pillar deflection curves according to the range of applied forces (blue). The deformation curve obtained using the experimental data for one pillar is shown in red. (F) Comparison of the experimental (red) and best-fitted simulated curve (blue) for one pillar indicates that the best match corresponds to an applied force of 230nN. The vertical green dashed lines indicate the horizontal force load domain. (G) Distribution of the forces exerted on the pillars by spheroids at day 4 after insertion in the microdevice (n = 342 micropillars from 21 microdevices with pillar diameter = 28 μm, and n = 172 micropillars from 12 microdevices with pillar diameter = 36 μm).

Then, we modelled the force/deflection relationship using FEM analysis (COMSOL). We considered each PDMS pillar as a cylinder, and used scanning electron microscopy and confocal microscopy to precisely measure the pillar geometrical dimensions. Microfabrication techniques allow controlling the pillar diameter, thus making possible to tune their spring constant (stiffness). For this study, we cultured spheroids in microdevices made of pillars of a constant height (300 μm), but with two different diameters (28 and 36 μm). We measured the PDMS Young’s modulus using the mechanical compression technique (E = 2.6 MPa). Then, we introduced these data in the model and used it for the mechanical simulation of deflection. To define where to load the force on the cylinder, we experimentally determined the contact zone between spheroid and pillars by measuring the height of the imprints left by the pillars on each MCF-7 spheroid after removal from the microdevice (after 4 days of culture) (Fig 3). We found that the contact zone corresponded to the upper 175 μm of the pillars (Fig 4A and 4B). The applied boundary force was considered horizontal and unidirectional because the growing spheroid within the microdevice is only confined on the sides by the pillars, whereas it is free on the top and the growth occurs quite homogeneously in all directions (as shown in S1 Movie). Using spheroids made with the mammary cancer cell line MCF-7, the observed pillar displacement was always outwards, therefore the force was simplified to horizontal according to the observation slice by slice of the pillar deformation that is fully compatible through simulations with an applied force normal to the pillar axis. Moreover, the applied boundary force was supposed to be uniformly distributed because this force is considered to mainly originate from the spheroid uniform growth, as the possible local mechanical efforts of individual cells (which can change from cell to cell) across the contact area could not be sensed.

Therefore, we uniformly applied a horizontal force load distributed over a rectangular area on the upper 175 μm of each 300 μm pillar (Fig 4B), and assigned a Dirichlet boundary condition to the bottom part of the pillar, meaning that the pillars are fixed on the support.

Force loading induced pillar deflection that could be extracted, and also Von Mises stress (Fig 4C and 4D). Moreover, we used a parametric sweep technique with applied forces ranging from 0 to 1000 nanoNewtons (nN) and a step of 10nN, and extracted the resulting range of simulated deflection curves (blue curves in Fig 4E and 4F).

Finally, to determine the forces generated by growing MCF-7 spheroids and responsible for pillar deflection, we compared the whole range of simulated deflection curves with the experimental deflection profile of each pillar in the microdevice, determined with the 3D reconstruction algorithm (red curve in Fig 4E and 4F). A MATLAB program allowed combining the results from the COMSOL simulations and the 3D reconstruction algorithm. We used spline interpolation for the COMSOL data to obtain the same discretization.

We computed three parameters (the sum of squares of residuals SS_res_, the coefficient of determination R^2^, and the largest shift M) to identify the simulated curve that best fitted the experimental curve for each 3D reconstructed pillar, using the equations described in the Methods section.

These parameters all provided similar results concerning the applied force responsible for the observed deformations (Fig 4F, only R^2^ is displayed as the most commonly used parameter). We then applied this method to quantify the force exerted on each pillar (either 28 or 36 μm in diameter) at day 4 after the beginning of the experiment. The measured forces ranged from 100 to 500 nN (Fig 4G) due to variations between pillars within one microdevice and between spheroids.

### Force increases with increasing pillar stiffness

To investigate whether pillar stiffness affected the forces generated by growing spheroids, we used microdevices made of pillars with a spring constant K = 8 nN/μm (pillars diameter = 28 μm) and K = 23 nN/μm (pillars diameter = 36 μm). In both microdevice types, spheroids growth was uniform and the surrounding pillars did not affect the 3D organization of the aggregates, according to our observations. Pillar deflection was smaller in microdevices made of stiffer pillars compared with softer pillars; however, the measured deflections for pillars with a high spring constant corresponded to higher applied forces. Indeed, the mean values of the measured forces using microdevices of K = 8 nN/μm and K = 23 nN/μm were F = 230±101 nN and F = 295±137 nN, respectively. These results show that spheroids exert higher forces when in contact with stiffer micropillars.

### Force changes over time

**Fig 5:**
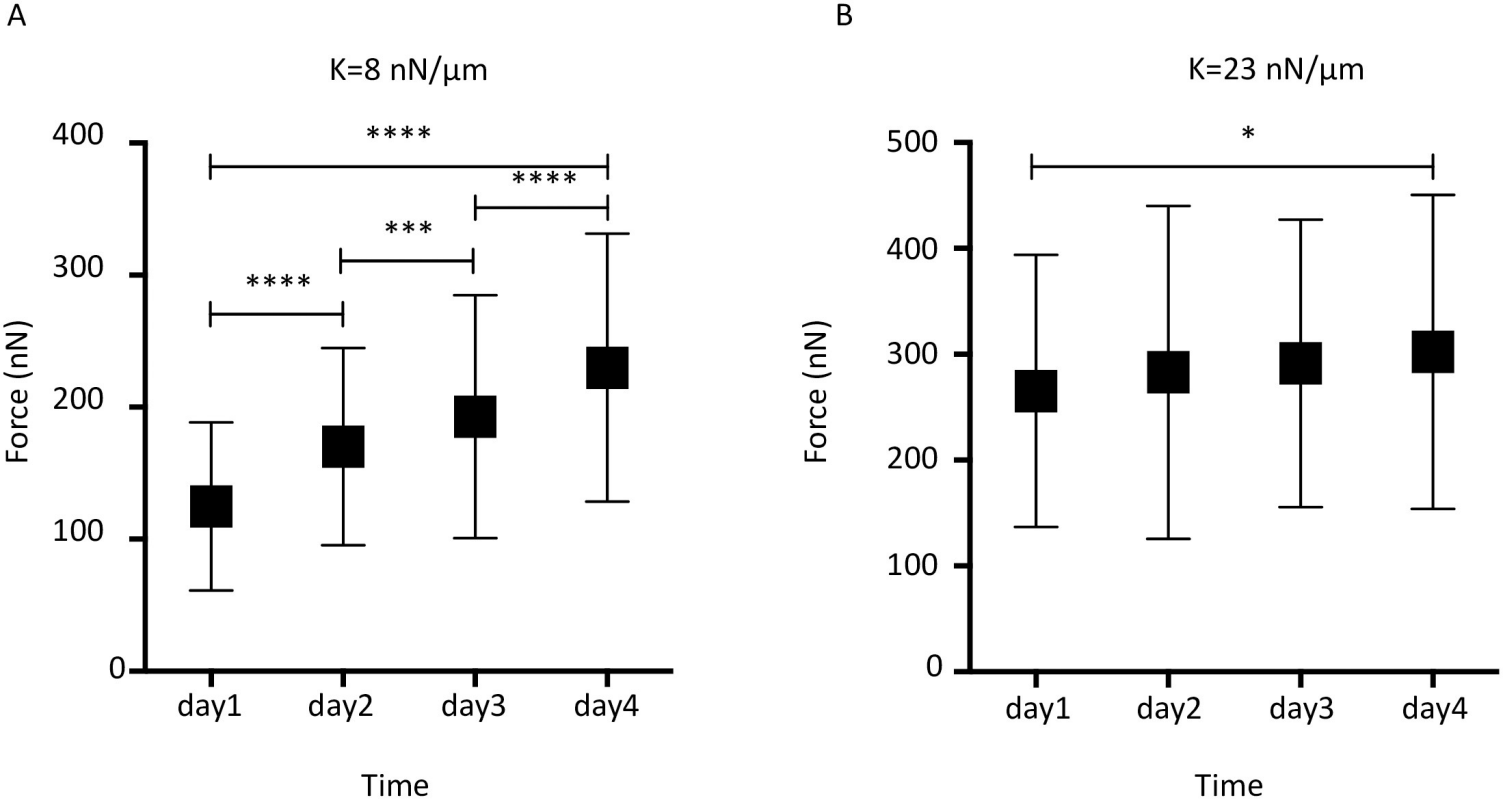
Monitoring of the detected forces induced by spheroid growth during four days. Forces detected using (A) microdevices with a pillar diameter = 28 μm and K = 8 nN/μm (n = 12, 16, 21, and 20 microdevices from day 1 to day 4), and (B) microdevices with pillar diameter = 36μm and K = 23 nN/μm (n = 8, 13, 10, and 12 microdevices from day 1 to day 4). Comparisons between days were performed using the two-tailed Student’s *t* test, 95% interval confidence: * = P <0.05, *** = P <0.001, **** = P <0.0001. Values are the mean ± standard deviation.

To better characterize the forces exhibited by growing spheroids, we investigated force changes over time. To that end, we monitored pillar deformation in a large number of microdevices during spheroid growth for four days. By comparing, as before, the experimental data obtained by volume imaging and 3D reconstruction with the simulation results, we could follow the changes in the forces generated by growing spheroids over time. For spheroids growing in the microdevices with soft pillars (K = 8 nN/μm), the mean values of the exerted forces progressively and significantly increased every day, from day 1 (121 nN) to day 4 (230 nN) (Fig 5A).

Conversely, for spheroids growing in devices with stiffer pillars (K = 23 nN/μm), the force increase (Fig 5B) was less important. Specifically, the mean value of the measured forces was 258 nN at day 1, which was higher than in devices with softer pillars; however, the exerted forces increased only of about 30 nN in four days. This corresponded to a rate of 0.42±0.01 nN/hr, which was more than three times lower than the rate observed in microdevices with softer pillars (1.40±0.16 nN/hr).

## Discussion and conclusion

We report here the investigation and characterization of the mechanical forces produced by growing multicellular tumour cell spheroids using microdevices made of high aspect ratio PDMS pillars that act as force sensors. Our approach is based on the experimental measurement of pillar deflection, assessed using 3D microscopy and reconstruction of the shape of the deformed device, as well as on FEM modelling to determine the forces responsible for the observed deformation.

The spheroid mechanical properties have also been investigated using atomic force microscopy measurements [18]. This technology is sensitive to the local mechanical surface properties of the cells at the spheroid periphery, and cannot be directly compared to the present work that was designed to characterize the mechanical pressure exerted by a growing micro-tumour.

Using pillars with a low spring constant (8 nN/μm), we found that forces exerted by a growing spheroid increased at a rate of around 33 nN/day, reaching a maximum of about 300 nN at day 4. These force values are higher than previously reported results on the forces exerted by single cells. For instance, force measurements in normal mammary epithelial MCF10A cells revealed a measured total force of 150 nN, which represents approximately 20 nN forces exerted on a single pillar with a 3.6 aspect ratio and a spring constant of 33 nN/μm [16]. The traction forces of an epithelial cell monolayer ranged between 5 and 20 nN (pillar spring constant of 21 nN/μm) and were concentrated on the edges, while single epithelial cells exerted forces between 2 and 3 nN [12]. Similarly, forces at single focal adhesions were in the order of 10 nN using fibroblasts [19]. The forces measured using spheroids are not traction forces, but mainly the result of cell proliferation and are roughly 10 times higher than traction forces measured for single cells and monolayers. When using microdevices with more rigid pillars (spring constant of 23 nN/μm versus 8 nN/μm), forces were higher from day 1, with a maximum force of about 400 nN. The pillar stiffness influence on force generation has been previously investigated in single cells with similar results. For instance, traction forces induced by fibroblasts were 16 nN and 41 nN when using micropillars with a spring constant of 12 nN/μm and 56 nN/μm, respectively, suggesting rigidity sensing [20]. Our observation strongly suggests that multicellular spheroids also sense and respond to mechanical stimuli (mechanosensing), the exact origin of which remains to be elucidated.

Other approaches have been developed to investigate the forces generated by spheroids based on traction force microscopy applied on spheroids embedded in a hydrogel or on spheroid encapsulation inside alginate capsules. Traction force microscopy in 3D revealed that unlike individual cells that exert traction forces, expanding spheroids exercise predominantly outward-directed tractions that are distributed over the surface of the multicellular structure [21]. These results are in line with the pillar radial outward displacement induced by the growth of MCF-7-derived spheroids within the microdevices described in our study. Compared with our results, the behaviour of spheroids in alginate capsules is quite different, probably because such spheroids reach confluence inside the capsule [3]. In this confined situation, the pressure exerted by spheroids drops and reaches a steady state, and cells at the spheroid periphery acquire a migrating phenotype. This situation is different from the growth of spheroids surrounded by pillars that do not lead to their confinement. Therefore, although pillars and alginate capsules are both mechanical sensors, it is difficult to compare pressure changes and rigidity sensing in these two experimental conditions.

The analysis of these results led us to propose a simple phenomenological analytical model for temporal changes of the force exerted by a growing spheroid (described in detail in Supplementary Information). This model is based on the hypothesis that force is the result of cell proliferation inside the spheroid, and assumes that the lateral confinement exerted by the surrounding pillars limits the spheroid volume increase. Such a simple model, developed with reasonable parameters, can be used to fit the experimental temporal changes in the spheroid growing forces and the dependence on the pillar stiffness (Supplementary Information).

In summary, we describe a methodology to evaluate the forces generated by growing spheroids based on the comparison of 3D imaging and reconstruction of the pillar deflection induced by spheroid growth with the simulation of pillar deflection. Using this method, we show that spheroids formed from a mammary cancer cell line (MCF7) generate higher forces against pillars of higher stiffness, suggesting that multicellular structures sense and respond to the properties of their mechanical environment. Moreover, by measuring the temporal changes of the force exerted by growing spheroids and by describing these modifications with a simple model, we deduce that the applied pressure reduces the cell division rate.

## Supporting information

S1 Movie Live imaging of a growing spheroid within a microdevice. Transmitted-light images from a time-lapse experiment (10X objective, 1 frame per 10 minutes) showing the growth of a MCF-7 spheroid within the microdevice.
(M4V)

S1 File Supporting information. This file includes detailed information on the 3D reconstruction algorithm and the presentation of a phenomenological model describing the temporal changes of the force exerted by a growing spheroid.
(PDF)
