# Supplementary material for: Measure and characterization of the forces exerted by growing multicellular spheroids using microdevice arrays
Source: PLoS One. 2019 May 23;14(5):e0217227. doi: 10.1371/journal.pone.0217227 (PMC6532909; doi:10.1371/journal.pone.0217227)
Supplement: S1 File — This file includes detailed information on the 3D reconstruction algorithm and the presentation of a phenomenological model describing the temporal changes of the force exerted by a growing spheroid. (PDF) [file pone.0217227.s002.pdf]

Supporting Information

3D reconstruction algorithm.

A crucial step to retrieve the forces exerted by spheroids was to reconstruct the pillar shape from the 3D images. This segmentation was achievable because: i) the number of pillars and their mean radius were known by design, ii) their shape was cylindrical, therefore pillar slices in z-planes look circular for low deflections. However, there were a few technical issues to be taken into account: i) the images were degraded by various optical defects (scattering, staining…) and the pillar slices were not simple disks (see figures A and B below), and ii) the pillar shapes could vary slightly across the z-planes. We therefore developed a simple and robust method to perform an automatic segmentation that takes into account these issues.

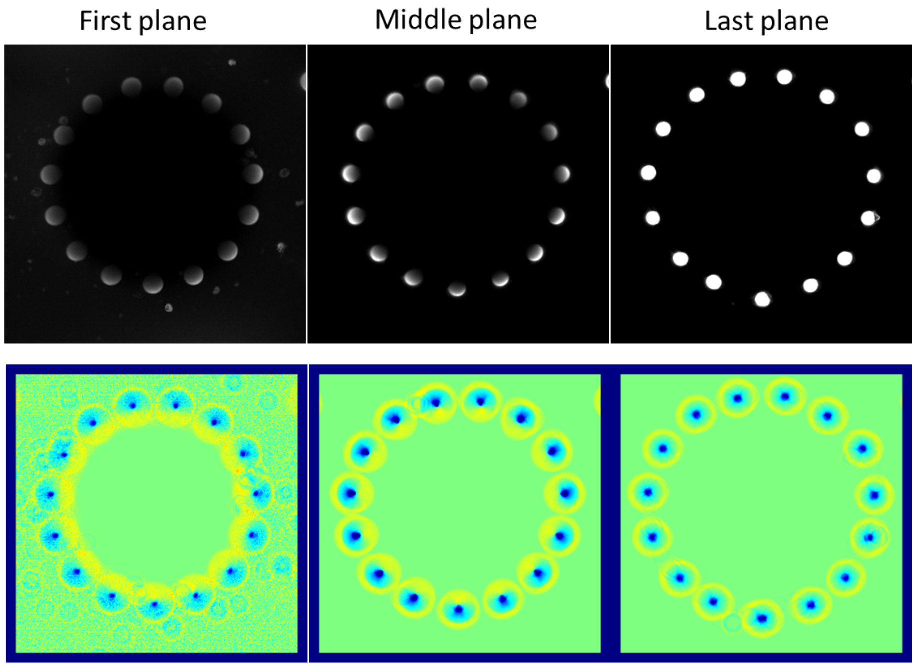

Figure A: Detection of the pillar radial displacement induced by MCF7 cell spheroids. The images represent the first, middle and last planes extracted from a z-stack with the fluorescently stained pillars (top) and the associated energy maps (bottom).

As mentioned in Materials and Methods, the algorithm works by sequentially segmenting each pillar, slice by slice. The general outline is as follows:

1- Segmentation of the pillar bases in 2D using the first slice of the 3D stack image.
2- From the second to the last slice: the information from the previous slice is used to define a search region and to segment the current slice.
3- Smoothing the pillar skeletons to reduce segmentation defects.

The algorithm outputs are the pillar centre and radius of each slice as well as a confidence index given by the minimization of an energy function.

Specifically, u denotes the 3D image, R_{min} and R_{max} denote the minimum and maximum pillar radii in pixels, and N denotes the pillar number. Let $B(\boldsymbol{x}_i, r_i)$ denote the Euclidean ball of radius r_i centered at location $\boldsymbol{x}_i = (x_i, y_i)$:

$$B(\boldsymbol{x}_i, r_i) = \{\boldsymbol{x} = (x, y) \in \mathbb{R}^2, \|\boldsymbol{x} - \boldsymbol{x}_i\|_2 = \sqrt{(x - x_i)^2 + (y - y_i)^2} \leq r_i\}.$$

For each slice, we formulated the segmentation problem as finding a set of non-overlapping disks that fit the image contents. The fit of a disk of centre $\boldsymbol{x}_i$ and radius r_i at slice z was defined through an energy (21) defined as follows:

$$E(\boldsymbol{x}_i, r_i, z) = \frac{1}{|\partial\omega|} \oint_{\partial\omega} \left\langle \frac{\nabla_{xy} u_\sigma(x,y,z)}{\sqrt{\left(|\nabla_{xy} u_\sigma(x,y,z)|^2 + \epsilon^2\right)}} \middle| \vec{n}(x,y) \right\rangle dl$$

where ω= $B(\boldsymbol{x}_i, r_i)$ is the disk and $\partial\omega$ is its boundary, $|\partial\omega|$ denotes the disk perimeter, u_σ is the image convolved with a Gaussian filter of variance σ, $\nabla_{xy} u$ denotes the gradient in the x-y plane, $\langle .|.\rangle$ denotes the Euclidean scalar product, $\vec{n}(x,y)$ denotes the outward normal to ω at a location $\boldsymbol{x} = (x,y)$ and ϵ is a regularization parameter that discards faint transitions. This energy has a few interesting features: i) it belongs to the interval [-1,1], -1 being the best fit and 1 indicating the worst one; ii) the convolution with a Gaussian filter ensures robustness to noise and other defects; iii) it is contrast-invariant, which basically means that the energy is invariant to the staining procedure and to the imaging device. This last feature is an essential ingredient that explains the method success. Figures B and D show the behavior of the energy for a fixed radius; they represent two different types of pillar displacements caused by two spheroid types of spheroids made of MCF7 (mammary cancer cell line) and GM637 (normal fibroblast cell line) cells. The pillar centres are well localized as the local minima of the energy.

The segmentation problem at slice z could be formulated as the following global optimization problem:

$$\min_{\substack{x_i \in Z_i(z) \\ r_i \in [R_{min}, R_{max}] \\ \|x_i - x_j\|_2 \leq r_i + r_j}} \sum_{i=1}^{N} E(\boldsymbol{x}_i, r_i, z),$$

where $(\boldsymbol{x}_i, r_i)_{1\leq i\leq N}$ are the disk centres and radii, and $Z_i(z) \subseteq \mathbb{R}^2$ denotes a search space for the i-th disk center. The constraints $\|\boldsymbol{x}_i - \boldsymbol{x}_j\|_2 \leq r_i + r_j$ are necessary and sufficient to ensure that the disks do not overlap. Unfortunately, the search space of this optimization problem is huge and global optimization methods could not provide satisfactory results in decent times. Therefore, we resorted to a slightly simpler method, where the sets $Z_i(z)$ are defined in such a way that their r_i-enlargement, defined by $Z_i^{r_i}(z) = \cup_{x\in Z_i} B(\boldsymbol{x}, r_i)$, satisfies $Z_i^{r_i}(z) \cap Z_j^{r_j}(z) = \emptyset, \forall i \neq j$. Under this assumption, the constraint $\|\boldsymbol{x}_i - \boldsymbol{x}_j\|_2 \leq r_i + r_j$ is automatically satisfied and each disk can be found independently of the others, breaking the curse of dimensionality.

We assumed that the 3D image contains n_z slices. We assumed without loss of generality that their location corresponded to the integer values$\{1, \dots, n_z\}$. The algorithm is divided into the three steps as follows:

Step 1. As the molds have a known geometry, it is easy to specify pre-defined search zones $Z_i(1)$. The segmentation problem is then solved to retrieve the disk locations and radii at slice 1.

Step 2. A simple method to define the next search zone consists in setting $Z_i(k+1) = B(\boldsymbol{x}_i(k), r)$, where r is a scalar describing the extent of the search zone. Unfortunately this

choice does not comply with the condition $Z_i^{r_i}(z) \cap Z_j^{r_j}(z) = \emptyset, \forall i \neq j$ unless the centres $\boldsymbol{x}_i(k)$ are well separated. We therefore resorted to front propagation algorithms to generate a sequence $(Z_i^l(k+1))_l$ starting with $Z_i^0(k+1) = B(\boldsymbol{x}_i(k), r)$ and to remove progressively pixels of the boundary until the non-overlapping condition is satisfied. Examples of pillars that are well segmented are presented in Figure C (note the red circles that are well superimposed with the pillars). The segmented pillars were numbered to confirm the right pairing of pillars from one image to the other when pillars are separated, or in contact (Figure C).
Step 3. Once slice segmentation is completed, discretized skeletons are obtained for each pillar given by the sequence $(\boldsymbol{x}_i(k))_{1 \leq k \leq n_z}$. These skeletons usually look a bit erratic due to imperfections of the segmentation (see left image in Figure E). Therefore, we smooth them using a polynomial approximation. The polynomial is optimized to minimize an l^2-norm weighted by the confidence level $E(\boldsymbol{x}_i(k), r_i(k), k)$. A result of these optimizations is presented in Figure E for the experiment on the pillars displaced by a MCF7 spheroid.

For an experiment with 55 images (size: 512x512 pixels) and 15 pillars (diameter: 25 pixels), the computing time of the MATLAB code is 75 seconds for 0.25 pixels precision. The portable computer used had a 2.4 GHz IntelCorei5 with 8 GB of RAM. The computing time could be easily improved by switching to a C++ implementation or computing a parallelized code on the graphic card.

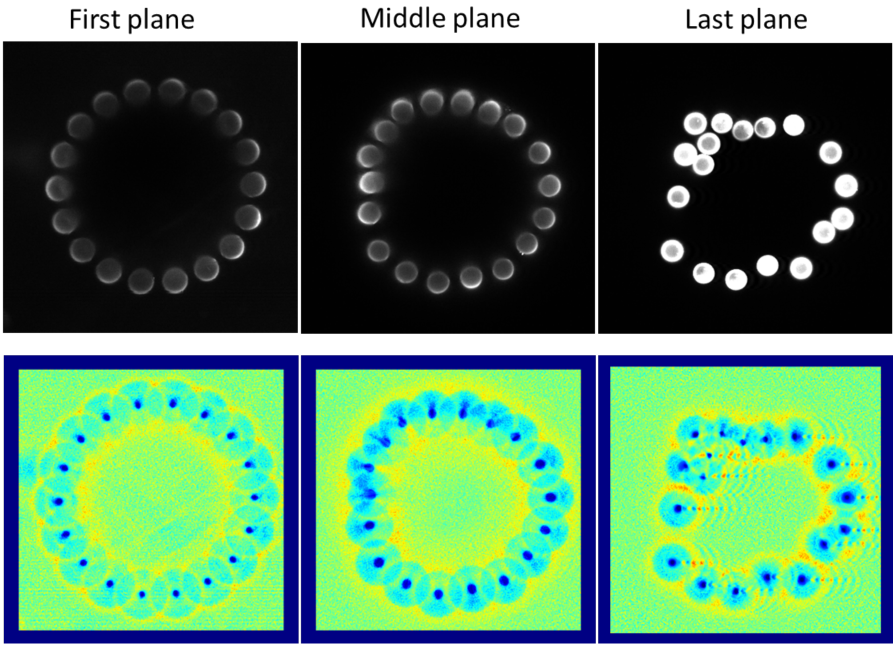

Figure B: Detection of pillar displacement induced by GM637 cell spheroids. Pillars came into contact with the spheroid towards the top of the microdevice. The images represent the first, middle and the last planes extracted from a z-stack of the fluorescently stained pillars (top) and of the associated energy maps (Bottom). The mechanical forces exerted by these spheroids are not discussed in this paper.

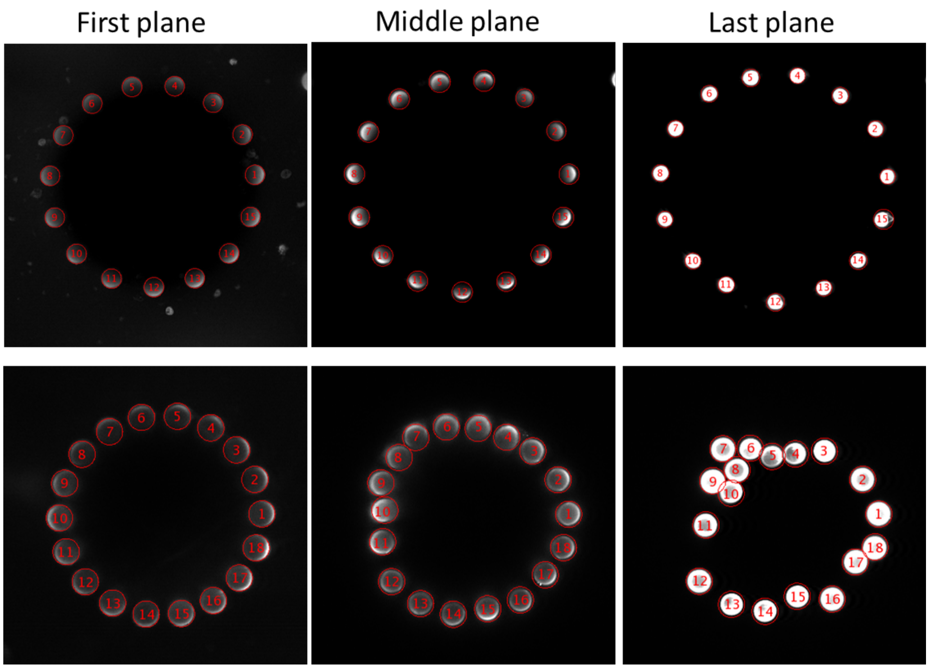

Figure C: 3D segmentation of pillar displacement. Accurate pillar segmentations are shown by the surrounding red circles, with conservation of the pillar order from one image to the other (as indicated by their numbering), shown on three levels of the z-stack images (first, middle and last). This procedure was used to monitor pillars displaced by a MCF7 cell spheroid where all the pillars are separated (top), and on pillars displaced by a GM637 cell spheroid where pillars are in contact in the last plane (bottom).

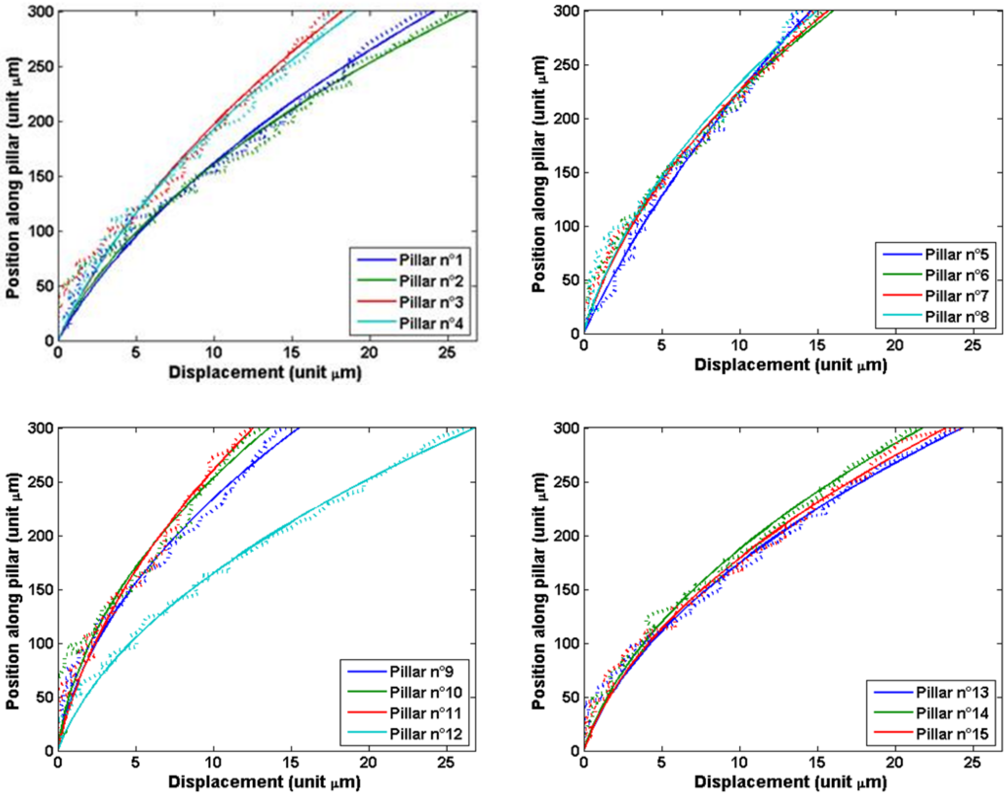

Figure D: Pillar deformation curves. Deformation curves for the 15 pillars displaced by the MCF7 cell spheroid shown in Figure A. The deformation curves for these pillars show the deformation magnitude along the 300µm length of each pillar, before (dots) and after smoothing (curves).

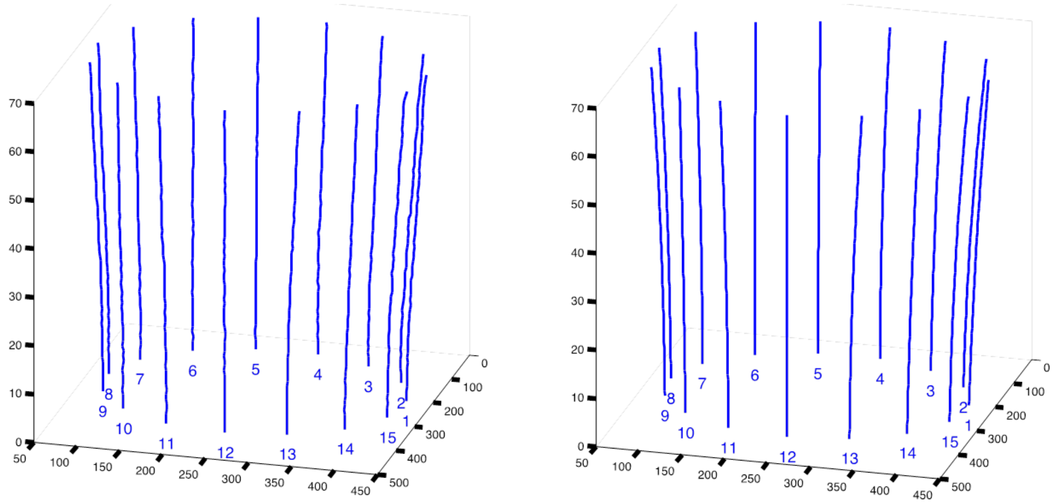

Figure E: 3D reconstruction of pillar displacement. This figure shows a 3D representation of the centre of all pillars of the microdevice from figure A, in each slice of the z-stack (75 slices with a z step of 4µm) before (left) and after smoothing (right).

Phenomenological model to describe the temporal changes of the force exerted by a growing spheroid.

Our objective was to propose a simple and phenomenological model of the confined growth of a spheroid inside a corral of pillars, in order to predict the temporal changes of applied forces due to spheroid growth.

Main hypothesis of the model

The main hypothesis of the model is that the force originates from the cell divisions occurring inside the spheroid (Figure F). As the spheroid is strongly confined laterally by the PDMS pillars, we made the approximation that despite the ongoing cell division and growth, the spheroid volume (V) did not expand much (ΔV was small) and pillar deflections in the order of a few microns were neglected. In the present model, we also did not include the possible growth and volume expansion in the vertical direction which give rise to an anisotropic growth (due to the strong lateral confinement exerted by the pillars). This restricts the domain of validity of our description to the first few days of culture during which the vertical spheroid growth remains limited.

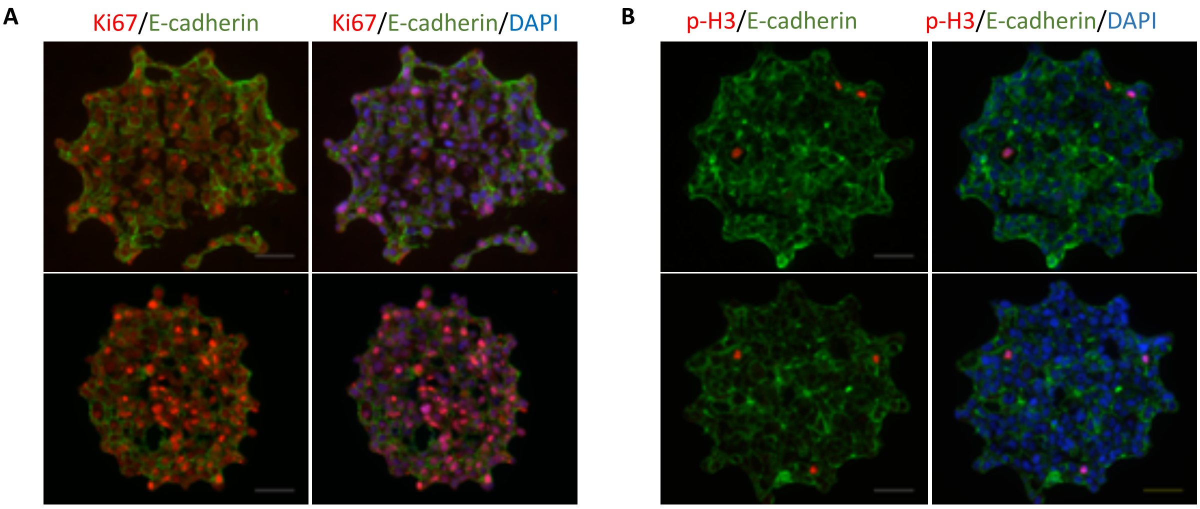

Figure F: Cell proliferation within spheroids after 4 days of growth in the microdevice. A. Immunodetection of the proliferation marker Ki67 and of the cell junction protein E-cadherin in cryosections from two different spheroids. B. Immunodetection of the mitotic marker phosphorylated histone H3 (p-H3) and of E-cadherin in cryosections from two different spheroids. Nuclei are stained with DAPI. Scale bar: 100µm.

Analytical development of the model

If the spheroid volume is kept constant and if cell division occurs at a rate k, k is the number of divisions by unit of time, then this means that the cell density ρ changes.

$\rho = \frac{N}{V}$

Where N is the total number of cells in the spheroid, and V is its volume.

Upon growth, cells in the spheroid get compressed, and this compression generates a deflection of the PDMS pillars and a cell density change.

If the volume expansion is negligible due to the strong lateral confinement, then we have:

$\frac{d\rho}{\rho} = \frac{dN}{N}$

If we now assume that as already proven [1], cell division occurs at the spheroid periphery whereas cells in the spheroid centre are quiescent, and we neglect cell death inside the spheroid, we have:

$\frac{dN}{dt} = k \times N_s$

Where N_s is the number of dividing cells in the spheroid outer region. Then,

$$N \times \frac{d\rho}{\rho} = k \times N_s \times dt$$

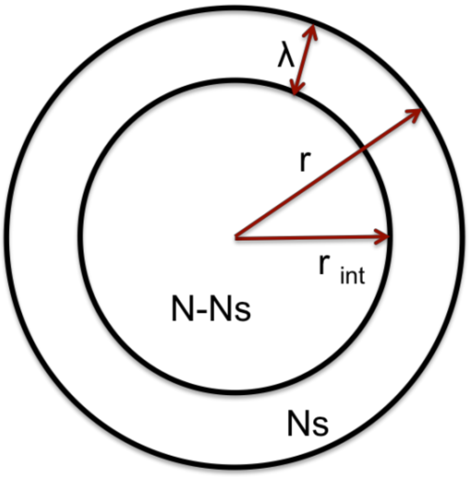

Figure G. Sketch of the spheroid parameters required for the model. The parameters are as follows: r is the radius of the whole spheroid, r_{int} is the radius of the necrotic core, λ is the thickness of the proliferating layer, N is the total number of cells, and Ns is the number of proliferating cells in the spheroid outer region.

Following previous authors [2,3,4], we introduced the compressibility modulus of the spheroid K, such as :

$$K = \rho \times \frac{dP}{d\rho}$$

where dP is the variation of the exerted pressure on the spheroid and dρ is the cell density variation. This parameter describes how the cell density changes (increases) when the pressure increases. The higher K is, the higher is the required pressure for changing cell density and the lower cell compressibility is. The lower K is, the lower the pressure required for changing cell density and the more compressible cells are. We thus have:

$$\frac{dP}{dt} = \frac{K \times k \times Ns}{N}$$

Considering spherical objects, if λ is small compared with r, then $\frac{Ns}{N} = \frac{3\lambda}{r}$ and:

$$\frac{dP}{dt} = \frac{K \times k \times 3\lambda}{r}$$

If we now consider that the pressure exerted on the spheroid is produced by the PDMS pillars, then the force sensed by the deformable pillars changes upon time, such as:

$$\frac{dF}{dt} = \frac{K \times k \times 3\lambda \times S}{r}$$

where S is the characteristic contact surface between one pillar and the spheroid.

It is straightforward to show that the quantity k.λ represents the growth rate of the radius of a free growing spheroid,

$$\frac{dr}{dt} = k \times \lambda = v$$

v being the growth rate of the radius of a freely growing spheroid. In fact this parameter, which is easily accessible experimentally, gives k, the cell division rate, thus:

$$\frac{dF}{dt} = \frac{3 \times K \times v \times S}{r}$$

However, as cells are compressed, they should have more and more difficulties to divide because this requires energy to create a free volume for the new cell. This energy can be expressed roughly as: $\Delta E = P x V_{cell}$, where P is the compressive stress and V_{cell} is the volume of a cell. This decrease of k with increasing compression can be phenomenologically introduced in the previous equation by considering that v is no more the growth rate of the radius of a free spheroid but the growth rate of a compressed spheroid v*. We assumed a linear dependence for this parameter with the compression force: $v^* = v - \alpha F$, where v is the growth rate of the radius of a freely growing spheroid, and α is a parameter indicating the decrease of growth rate (cell division rate) upon a compressive force F. The temporal changes of the force sensed by the pillars during the spheroid growth can be written as:

$$F(t) = \frac{v}{\alpha}\,(1 - e^{\frac{-3 \times K \times S \times \alpha \times t}{r}})$$

Finally, to include a complete description of the experimentally observed phenomena, we postulated that the compressibility K of the spheroid depends on the stiffness of the confining structures. Indeed, it has been shown that cells sense the stiffness of their environment and exert higher forces on stiffer substrates. This mechanosensing effects clearly suggests that if cells are in contact with a stiffer environment, they will become more contractile, and thus less easily compressible. This translates into an increase of the parameter K.

The model gives the following predictions:

- The force saturates with time. This prediction is difficult to check experimentally because before reaching this saturation, the spheroid will start to exhibit an anisotropic growth, preferring to expand in the vertical direction, and this effect is not taken into account by our model. This means that the domain of validity of our model is restricted to the initial growth period, well before approaching the saturating force.
- The time increase of the forces in the initial linear regime ($3KS\alpha/r$) is higher for stiff pillars than for soft pillars due to the change of K as a function of the stiffness of the micro-environment.

Orders of magnitude

-The free growth rate of the radius of our spheroids was measured experimentally and is $v = 3.10^{-10}$ m/s.
-According to previous studies, the cell compressibility K is around 1KPa [2,3,4].
-The radius of the investigated spheroids (device) is r=125µm.
-The maximal measured force at saturation is around 300nN.
- The surface of contact between the spheroid scales as r_{pillar}^2, giving $S \approx 2.10^{-10}$ m^2.
- The value of α, deduced from the maximal force (300nN), is $\alpha \approx 10^{-3}$ $m.s^{-1}.N^{-1}$

As shown in Figure H, the model fits reasonably well the experimental points, demonstrating that the orders of magnitude are in agreement with our experimental data and that our model describes the most salient phenomena observed experimentally.

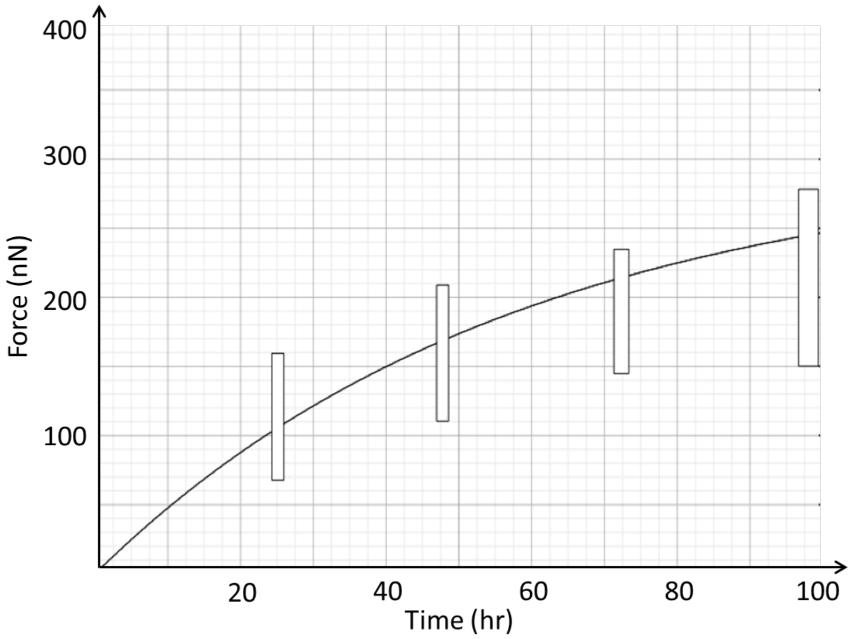

Figure H. Temporal force changes as given by the analytical model. The model takes into consideration K (cell compressibility), S (surface of contact between the spheroid and one pillar), r (spheroid radius), v (growth rate of the spheroid radius), and α (decrease of the growth rate in the presence of a compressive force). The white rectangles show the experimental measurements obtained with soft pillars (diameter 28 µm) accounting for the dispersion of the measured forces at the indicated time points.

A recent publication [5] showed that high pressures exerted on spheroids inhibit cell proliferation. In this article, the division rate k decreased linearly with the applied pressure P, in the range of 0-10 KPa. From the curves shown in this study, the slope β of the decrease of k with P can be estimated to be around β=dK/dP= 10^{-9}- 10^{-10} (s^{-1} Pa^{-1}). In our model, we introduced a parameter α that represents the linear decrease of the growth radius velocity with the applied force (dv/dF= α). As $k \times \lambda = v$ and F/S=P, it is possible to compare the value of α extracted from our data with the β value obtained in [5]. By transforming α =dv/dF in β=dK/dP and assuming S=2.10^{-10} m^2, α=10^{-3} m.s^{-1}.N^{-1}, λ=100 µm, we obtained β=2x10^{-9} (s^{-1} Pa^{-1}).
This value is quite comparable to the one given in [5], showing that probably the two experiments, although very different, characterize the same effect: the decrease of cell division rate under pressure.

In conclusion, we think that although based on crude approximations, our phenomenological model can capture all the observed experimental data and is in reasonable agreement with the order of magnitude of the involved parameters.

References

[1] Delarue M, Montel F, Vignjevic D, Prost J, Joanny JF, Cappello G. Compressive stress inhibits proliferation in tumor spheroids through a volume limitation. Biophysical journal. 2014 Oct 21;107(8):1821-8. PubMed PMID: 25418163. Pubmed Central PMCID: 4213738.

[2] Drasdo D, Hohme S. A single-cell-based model of tumor growth in vitro: monolayers and spheroids. Physical biology. 2005 Sep;2(3):133-47. PubMed PMID: 16224119

[3] Lin L, Liu A, Yu Y, Zhang C, Lim C, Ng S, et al. Cell compressibility studies utilizing noncontact hydrostatic pressure measurements on single living cells in a microchamber. Applied Physics Letters. 2008;92(23):233901.

[4] Alessandri K, Sarangi BR, Gurchenkov VV, Sinha B, Kiessling TR, Fetler L, et al. Cellular capsules as a tool for multicellular spheroid production and for investigating the mechanics of tumor progression in vitro. Proc Natl Acad Sci U S A. 2013 Sep 10;110(37):14843-8. PubMed PMID: 23980147. Pubmed Central PMCID: 3773746.

[5] Montel F, Delarue M, Elgeti J, Malaquin L, Basan M, Risler T, et al. Stress clamp experiments on multicellular tumor spheroids. Physical review letters. 2011 Oct 28;107(18):188102. PubMed PMID: 22107677.
